# The emerging role of MIR-146A in the control of hematopoiesis, immune function and cancer

**DOI:** 10.1186/1756-8722-5-13

**Published:** 2012-03-27

**Authors:** Catherine Labbaye, Ugo Testa

**Affiliations:** 1 Department of Hematology, Oncology and Molecular Medicine, Istituto Superiore di Sanità, Viale Regina Elena 299, 00161 Rome, Italy

## Abstract

MicroRNA (miRs) represent a class of small non-coding regulatory RNAs playing a major role in the control of gene expression by repressing protein synthesis at the post-transcriptional level. Studies carried out during the last years have shown that some miRNAs plays a key role in the control of normal and malignant hgematopoiesis. In this review we focus on recent progress in analyzing the functional role of miR-146a in the control of normal and malignant hematopoiesis. On the other hand, this miRNA has shown to impact in the control of innate immune responses. Finally, many recent studies indicate a deregulation of miR-146 in many solid tumors and gene knockout studies indicate a role for this miRNA as a tumor suppressor.

## MiR-146a: discovery and targets

Most vertebrates have two copies of the gene encoding miR-146, miR-146a and miR-146b, located at the level of different chromosomes, 5 and 10, respectively, within different genes. The structural differences of these two miRNAs are very limited since they differ in their mature sequences by just two nucleotides located at the 3' end. In spite these great structural similarities, miR-146a and miR-146b do not seem to be redundant in their biologic activity, as suggested by the observation that they may have a different post-transcritpional processing mechanism: thus, following lipopolysaccharide stimulation the transcription of both miR-146a and miR-146b is stimulated, but only mature miR-146a is produced [1].

MiR-146a was initially discovered during a systematic study aiming to identify miRNAs that play a potentially important role in the innate immune response to microbial infection [2]. Particularly, expression profiling of 200 miRNAs in human monocytes revealed that some miRNAs, including miR-146a and miR-155, are strongly upmodulated following challenging of these cells with bacterial endotoxin [2]. The analysis of the miR-146a promoter provided evidence that miR-146a is a NF-kappaB-dependent gene [2]. miR-146a was predicted to base-pair with sequences in the 3' UTR of the TNF receptor-associated factor 6 (TRAF6) and IL-1 receptor-associated kinase 1 (IRAK1) genes (Table 1). Both these two genes encode key adaptor molecules downstream of Toll-like and cytokine receptors.

**Table 1:** Molecular Targets of miR-146a

*Target Molecules*	*Cellular System*	*Biological Consequences*	*References*
TRAF6, IRAK1 (adaptor molecules downstream of Toll-like and cytokine receptors)	LPS-stimulated monocytes	Innate immunity response	Taganov et al, PNAS, [2]

IRAK2 (adaptor molecules downstream of Toll-like and cytokine receptors)	VSV-infected macrophages	Innate immunity response	Hou et al, J Immunol [3]

IL-8, RANTES	Lung Epithelial Alveolar Cells	Innate immunity response	Perry MM et al, J Immunol [4]

CCL((MCP2)	HIV-infected microglial cells	Innate immunity response	Rom et al, FASEB J, [5]

FADD	Activated T lymphocytes Jurkat cells	Anti-apoptotic effect Adaptive immune response	Curtale et al, Blood [6]

EGF-R	Breast, pancreatic and gastric cancer	Cell proliferation, survival	Hurst et al, Cancer Res [7] Li et al, Cancer Res, [8] Kogo et al, Clin Cancer Res, [9]

ROCK1	Prostate cancer cells	Cell proliferation, invasion and metastasis	Lin et al, RNA, [10]

NOTCH1	Glioblastoma	Cell proliferation, survival and differentiation	Mei et al, Mol Cell Biol, [11]

CXCR4	Leukemic cell lines Hematopoietic progenitors induced to megakaryocytic differentiation Kaposi's sarcoma associated herpesvirus-infected cells	Cell migration, proliferation and differentiation	Labbaye et al, Nat Cell Biol, [12]

KLF4	Vascular smooth muscle cells	Vascular muscle proliferation	Sun et al, EMBO Rep, [13]

Studies carried out in vesicular stomatitis virus (VSV)-infected macrophages showed that macrophage infarction induced a marked upmodulation of miR-146a that was shown to target IRAK2, in addition to the previously reported TRAF6 and IRAK1 [3].

The study of human lung alveolar epithelial cells have led to the identification of additional miR-146a targets involved in the control of innate immunity. In fact, Perry and coworkers showed that exposure of human lung alveolar epithelial cells to IL-1β resulted in a very pronounced increase of miR-146a levels, that in turn induced a decrease of IL-8 and RANTES chemokines through targeting of the 3' UTRs of their respective mRNAs [4].

Based on experiments carried out on HIV-infected microglial cells, Rom et al have shown that miR-146a targets in these cells the 3' UTR of the CCL8 (MCP2) mRNA, leading to a decreased release of this chemokine [5]. According to these observations it was suggested that miR-146a may act as a modulator in macrophages/microglial cells of the production of the pro-inflammatory cytokine CCL8 (MCP2).

A recent study provided evidence that miR-146a, in addition to act as a modulator of the innate immune response, plays also a role as a modulator of the adaptive immune response. Thus, it was shown that miR-146a is scarcely expressed in naïve T cells, while is abundantly expressed in human memory T cells [6]. miR-146a expression is clearly induced following T cell activation via T cell receptor stimulation. During T cell activation miR-146a upmodulation plays an anti-apoptotic role, mediated through the targeting of FADD mRNA [6]. miR-146a modulates also IL-2 production in activated T lymphocytes [6]. According to all these observations it was suggested that miR-146a plays also a role in the modulation of adaptive immunity.

In addition to these targets all involved at various levels in the mechanisms of innate immunity, miR-146a targets also other mRNAs encoding for proteins whose function in more related to the controls of other cellular activities, such as cell proliferation, differentiation and migration. Thus, studies carried out on breast cancer cells have led to identify EGF-R as a possible target of miR-146a (Table 1). These studies were originated from initial observations showing that overexpression of miR-146a in breast cancer cells determines an inhibition of NF-kB activity with reduction of metastatic potential [14]. After this initial observation, in a second study it was shown that the capacity of the Breast Cancer Metastasis Suppressor 1 (BRMS1) protein was dependent on miR-146a upregulation that in turn reduces the metastatic potential of breast cancer cells and was associated with a downmodulation of the Epidermal growth Factor Receptor [7]. These findings were confirmed in pancreatic cancer cells showing that natural compounds, such as isoflavone, suppress the invasive properties of these cells through a mechanism which involves miR-146a upmodulation that in turn leads to a decline of EGFR expression [8]. Similar observations were recently reported also in gastric cancer cells, where it was shown that enforced expression of miR-146a inhibited migration and invasion of tumor cells and downregulated EGFR and IRAK1 expression [9]. Importantly, this study showed also that the miR-146a downmodulates EGFR expression through a direct mechanism involving targeting of the 3' UTR of the EGFR mRNA [9]. In cardiomyocytes miR-146a targets ERB4, another member of the EGFR family; in these cells miR-146a up-regulation was related to cardiotoxic events [15].

A recent study provided some evidence that miR-146a overexpression in glioblastomas exerts an anti-tumor effect, seemingly related to the capacity of this microRNA to target in these cells the 3' UTR of the NOTCH1 mRNA [11].

Studies carried out in leukemic cell lines and in normal megakaryocytes have led to the identification of a new target of miR-146a, the chemokine receptor CXCR4 [12] (Table 1). Particularly, the identification of this target was related to studies on PLZF, where it was shown that this transcription factor inhibits miR-146a expression and in turn up-regulates CXCR4 expression [12]. Through *in vitro *assays it was shown that PLZF is able to interact with the miR-146a promoter, while miR-146a targets the 3' UTR of the CXCR4 mRNA, impeding its translation [12]. During normal megakaryocytic differentiation a functionally regulatory loop was observed, involving PLZF suppressing miR-146a transcription and thereby activating CXCR4 translation [12]. According to these studies, abnormalities of miR-146a expression (downregulation) in tumors may have important consequences for malignancy through enhanced CXCR4 expression in these cells. The targeting of CXCR4 mRNA by miR-146a was confirmed also by other studies showing that the Kaposi's sarcoma-associated hepesvirus-encoded viral FLICE inhibitor protein (v-FLIP) mediates CXCR4 suppression through miR-146a upmodulation [16].

Additional miR-146a targets have been recently identified. In vascular smooth muscle cells miR-146a targets the 3' UTR od Kruppel-like Factor 4 (KLF4): silencing of miR-146a increases KLF4 expression, while its overexpression induces an opposite effect [13] (Table 1). In turn, KLF4 acts as a regulator of miR-146a transcription, acting as a repressor of miR-146a transcription [13]. The biological consequences of this modulation of KLF4 exerted by miR-146a consist in a stimulation of vascular muscle proliferation *in vitro *and a vascular neointimal hyperplasia *in vivo *elicited by miR-146a overexpression. In prostate cancer cells ROCK1, one of the key kinases for the activation of the hyaluronan, was found to be targeted by miR-146a [10]. miR-146a enforced expression in these cancer cells leads to a marked downmodulation of ROCK1, associated with a reduced proliferation, migration and invasiveness [10].

## Expression and function of miR-146a in immune cells

Many studies were conveyed to the analysis of the expression and function of miR-146a in various cell types of the immune system. The analysis of miR-146a expression in highly purified subsets of cells of the immune system showed that miR-146a is among the few miRs to be differentially expressed between Th1 and Th2 cells in the mouse, thus suggesting that it could be involved in fate determination of these cells. Studies in mice made deficient in miR-146a expression by gene knockout showed an increase in the number of IFNgamma-producing T-cell subset (Treg) [10]. In spite the increased number of Treg lymphocytes induced by miR-146a deficiency, their function resulted to be impaired, with a consequent breakdown of immunological tolerance with massive lymphocyte activation and tissue infiltration in several organs [17]. In human T lymphocytes, miR-146a is scarcely expressed in naïve T cells, while it is abundantly expressed in memory T cells and is induced upon TCR stimulation [6].

As mentioned above, miR-146a is a typical miRNA involved in the control fo the inflammatory response of cells of the innate immune system and, particularly monocytes/macrophages, to infection. As mentioned in the previous section, miR-146a was found to be inducible in human monocytes upon stimulation with LPS in a NF-kB dependent manner and to target TRAF6 and IRAK1 mRNAs. These two genes encode key adaptor molecules acting downstream of TLRs and cytokine receptors, suggesting a role for miR-146a in controlling signaling originated from the activation of these receptors through a negative feedback loop involving downregulation of TRAF6 and IRAK1.

Other studies suggest an important role for miR-146a in the development of the mechanism of LPS tolerance. The LPS tolerance is a peculiar property of monocytes to develop a condition of hyporesponsiveness to LPS (with reduced TNF-alpha production) upon prolonged exposure to LPS. This conclusion was based on the observations that miR-146a continues to increase over 24 h exposure to LPS, while TNF-alpha levels increased during the first 4 h and then progressively decreased [18]. Transfection of a miR-146a inhibitor in THP-1 cells inhibited the development of a LPS tolerogenic response [18]. A role for miR-146a was also shown in the phenomenon of LPS-induced cross-tolerance, i.e., a mechanism of tolerance to both homologous and heterologous inflammatory ligands [19]. miR-146a could therefore act as a fine tuning mechanism to prevent an overstimulation of the inflammatory response.

MiR-146a expression, explored in dendritic cells, is about 6-fold more highly expressed in Langheran's cells than in interstitial dendritic cells generated in vitro from human granulocyte/myeloid progenitor cells [20]. Importantly, miR-146a expression, at variance with the findings observed in monocytes, does not increase in response to cell activation, thus suggesting that in Langheran's cells miR-146a expression is constitutively high. The high expression of miR-146a in Langheran's cells seems to be induced by the transcription factor PU.1 [20]. According to these observations it was suggested that elevated miR-146a expression could be required for Langheran's cells development and could prevent in these cells inappropriate cell activation resulting from environmental Toll-like receptor ligands.

## Role of miR-146a in cell differentiation

Many recent studies indicate that miR-146a expression is finely tuned during cell differentiation and the miR-146a-mediated control of some targets plays in turn an important role in the process of cell differentiation. These studies were mainly focused to analyze the effect of miR-146a on hematopoietic cell differentiation.

Some evidences indicate a role for miR-146a in the control of macrophage differentiation. Ghani et al using a model of PU.1 inducible cell lines showed that activation of the expression of this transcription factor induced a marked upmodulation of miR-146a expression [21]. Stimulation of miR-146a expression by PU.1 is mediated by a transcriptional stimulation dependent upon the binding of PU.1 to a binding motif located at 10 kb upstream of the miR-146a locus. miR-146a was found to be expressed at low level in purified preparations of murine common myeloid progenitors and at markedly higher levels in mature myeloid cells and particularly in macrophages. Loss of function experiments in murine and zebrafish models revealed that generation of the macrophage compartment in embryos requires the regulatory activity of miR-146a [21]. According to these findings it was suggested that miR-146a plays an important role not only in inhibition of pro-inflammatory signaling, but also in promoting macrophage development from hematopoietic stem cells [21].

The possible effects of miR-146a deficiency on myelopoiesis were evaluated in murine gene knockout models. Thus Boldin et al reported the knockout (KO) of miR-146a: as expected, monocytes derived from miR-146a-null mice were hypersensitive to LPS [22]. Although no overt abnormalities of hematopoietic differentiation have been observed in miR-146a-deficient mice, abnormalities of some hematopoietic lineages were found. Thus miR-146a^-/- ^mice showed a marked splenomegaly due to the accumulation and increased proliferation of myeloid elements CD11b^+^/Mac1^+ ^[22]. Aging miR-146a KO mice displayed the development of anemia, thrombocytopenia and lymphopenia, seemingly in consequence of myeloproliferative syndrome [22]. The proliferation of these myeloid elements seems to be in part due to the overexpression of M-CSFR [22]. However, the cellular lineage of the myeloproliferation and the mechanistic basis of miR-146a deficiency-mediated myeloproliferation remained to be evaluated. Thus, in a second study the same authors showed that miR-146a KO mice developed myeloid sarcomas through a mechanism mediated via NF-kB activation induced by miR-146a deficiency [23].

These observations indicate that, in addition to its anti-inflammatory role, miR-146a plays a negative role in the development of myeloid elements since its deletion in mice results in a myeloproliferative disorder with accumulation of CD11b^+^Mac1^+ ^cells.

In another study a different approach was attempted to explore miR-146a function in hematopoietic differentiation. Thus, miR-146a was overexpressed in hematopoietic stem cells, followed by bone marrow transplantation. The transplantation of these cells resulted in a transient myeloid expansion, decreased erythropoiesis and impaired lymphopoiesis [24]. Furthermore, the enforced expression of miR-146a impaired bone marrow reconstitutuion in recipient mice and reduced survival of hematopoietic stem cells [24]. It is also important to note that in this study miR-146a was found to be expressed mainly in primitive hematopoietic stem cells, lymphocytes and monocytes [24].

A number of recent studies support a role of miR-146a in the control of megakaryocytopoiesis. miR-146a levels have been reported to dramatically change during megakaryocytic differentiation of hematopoietic progenitor cells, but there is conflicting evidence regarding the direction of change and the function of miR-146a in megakaryocytic differentiation. Thus, Labbaye et al observed that miR-146a is abundantly expressed in human hematopoietic progenitors cells (HPCs) and its expression decreased when these cells are induced to differentiate to megakaryocytes [12]. Importantly, forced expression of miR-146a into HPCs caused a reduction of the maturation of megakaryocytic cells, as evidenced by a reduced expression of membrane megakaryocytic differentiation markers and a reduction in the number of polyploidy cells [12]. Conversely, inhibition of miR-146a expression by antagomir caused an opposite effect, with increased expression of megakaryocytic differentiation markers and increased polyploidy [12]. The reduction of miR-146a expression associated with megakaryocytic differentiation was confirmed also by Garzon et al [25] who observed miR-146a levels much higher in normal bone marrow (BM) CD34^+ ^cells than in BM megakaryocytes [25]. In line with these findings, Starczynowski et al showed that miR-146a was strongly downmodulated during hematopoietic differentiation since it was markedly more expressed in HPCs CD34^+ ^than in CD34^- ^BM precursor cells [26]. In their studies Starczynowski et al demonstrated that knockdown of miR-146a and miR-145 in murine HPCs (by overexpression of decoy targets or anti-miRNA) resulted after transplant in significant thrombocytosis and increased megakaryocyte colony formation [26].

In contrast to these findings, Opalinska et al reported that in murine HPCs induced to differentiate to megakaryocytres, the expression of miR-146a increased [27]. Furthermore, these authors showed that overexpression of miR-146a in mouse bone marrow HPCs produced no significant alterations in megakaryocyte development *in vivo *after transplantation and no changes in megakaryocyte colony assays [27].

The effect of miR-146a overexpression in murine HPCs was explored also by Starzynowski et al [24]. These authors showed that miR-146a is mainly expressed in stem/progenitor cells and that its overexpression in HPCs, followed by bone marrow transplantation, resulted in a number of hematological abnormalities consisting in transient myeloid expansion, decreased erythropoiesis and impaired lymphopoiesis; furthermore, miR-146a overexpression reduced bone marrow reconstitution capacity [24]. However, surpisingly, miR-146a overexpression did not apparently affect megakaryocyte number and differentiation [24]. It was proposed that these apparently conflicting results may derive from species differences and different experimental culture conditions that could result in different levels of functional miR-146a [28]. Particularly, it is possible that overexpression of miR-146a beyond endogenous levels could be not sufficient to induce a biological effect on megakaryocytes [29].

An additional great complexity of these studies is related to the mechanisms through which miR-146a could affect megakaryocyte development. Thus, Labbaye et al provided evidence that miR-146a controls megakaryocyte differentiation through a cell autonomous mechanism related to the targeting of the chemokine receptor CXCR4. Thus, the decrease of miR-146a expression observed during megakaryocytic differentiation permits the optimal expression of CXCR4 in developing megakaryocytes: the autocrine stimulatioin of this receptor by endogenously released Stromal derived Fcator-1 (SDF-1alpha) activates CXCR4 and its signaling together with TPOR stimulates megakaryocytic differentiation [12]. In contrast, Starczynovski and coworkers in their studies on miR-145/miR-146a-deficient mice suggested a different, non cell-autonomous mechanism, based on the targeting of TRAF6 by miR-146a; TRAF6 acts as a stimulator of IL-6, a cytokine required for optimal megakaryocytic proliferation/differentiation [26].

## miR-146a and cancer

Many microRNAs, including miR-146a, have been shown to contribute to the complex molecular mechanisms involved in the control of cell growth, differentiation and survival, processes mainly related to cancer development and progression (reviewed in 30). A large number of recent studies has shown the utility of microRNAs as cancer-related biomarkers, as supported by the finding that some microRNAs display altered expression profiles in cancers compared to normal tissues [30]. Finally, the altered expression of some of these microRNAs play a key role in tumor progression and therefore may represent targets for the development of new therapies [30].

Many recent studies have suggested a role for miR-146a in the development and maintenance of neoplastic processes.

Several studies carried out in papillary thyroid carcinoma were among the first studies to show abnormalities of miR-146 in some human cancers. Thus, He et al showed that several miRNAs are up-regulated in papillary thyroid carcinoma compared to normal thyroid tissue: among them, particularly pronounced was the upregulation of miR-146b, miR-221 and miR-222 [31]. Tumors in which the upregulation of these three miRNAs was strong showed a marked loss of kit protein, a target shared by all three miRNAs [31]. Subsequent studies provided evidence about a more complex link between deregulated miR-146 expression and thyroid papillary cancer development. Thus, Jazdewski et al showed the existence of a common G/C polymorphism within the pre-miR-146a sequence, reducing the amount of miR-146a produced by the involved allele (C allele) by about 2 fold, compared to the wild-type allele [32]. The reduced production of miR-146a by the C allele leads to a reduced downmodulation of the corresponding target genes. A screening carried out on a large number of patients with papillary thyroid carcinoma, compared to a similarly large number of normal controls showed that the GC heterozygote state, but not the homozygote CC state, is associated with an increased risk of acquiring papillary thyroid cancer; furthermore, 4.7% of the tumors had undergone mutations of the SNP sequence [32].

From this study it remained unclear why heterozigosity for the G/C polymorphism, but not homozygosity, in the pre miR-146a predisposes to papillary thyroid cancer. This point was clarified in a subsequent study showing that GC heterozygotes, but not GG and CC homozygotes, produce 3 mature microRNAs: one from the leading stand and two from the passenger strand (miR-146a*G and miR-146a*C), each with its distinct set of target genes [33]. To understand this point it must be taken into account that miRs are transcribed from DNA and progressively processed from primary transcript (pri-miR) to a hairpin precursor (pre-miR), comprising two strands: the leading strand used for the production of mature miR and the passenger strand that is commonly believed to be degraded. Therefore, the study of miR-146a showed that novel mature miR is produced from the passenger strand and the presence of SNP generates two isoforms of the alternative miR: miR-146a*G from the allele carrying G and miR-146a*C from the allele carrying C [33]. In a microarray expression study, the transcriptosomes of individual genotypes differed considerably, with the modulated genes mainly involved in the control of apoptosis [34]. This can determine an exaggerated DNA-damage response in heterozygotes, thus explaining the predisposition to develop thyroid cancer [34].

The increased expression of miR-146a in anaplastic thyroid cancer could be related to the high and spontaneous NF-kB activity observed in these tumor cells: in fact, it was shown that NF-kB stimulates miR-146a transcription [35].

Several studies were focused to explore the abnormalities of miR-146 expression in pancreatic carcinoma. miR-146a expression was found to be downregulated in pancreatic cells compared to the levels observed in their normal counterpart [36]. Re-expression of miR-146a in pancreatic cancer cells led to the inhibition of both EGFR and NF-kB signaling; this effect is paralleled by a reduced invasion capacity of these cancer cells [36]. Another study more directly focused to the comparative analysis of pancreatic tumor tissue and normal pancreatic tissue: thus, pancreatic intraepithelial neoplasms and pancreatic ductal adenocarcinomas differentially expressed 35 miRNAs compared with normal pancreatic ductal tissue and, among them, one of the most downmodulated was miR-146a [37]. A rescue of miR-146a expression in pancreatic cancer cells could contribute to the inhibition of the malignant phenotype. Thus, Bao et al have shown that curcumin inhibits pancreatic tumor growth by attenuating the expression of the histone methyl-transferase E2H2 and increasing the expression of a panel of tumor suppressor miRNA, including miR-146a [38].

miR-146a levels have been explored in a large set of gastric cancer patients and resulted to be significantly lower in cancerous than in noncancerous tissue [9]. Furthermore, the subdivision of these 90 gastric cancer patients into two subgroups, i.e., those exhibiting high and those exhibiting low miR-146a levels, showed that the low-expression miR-146a group exhibited more extensive lymph node metastasis and venous invasion than the high-expression group and, importantly, a significantly poorer prognosis, as evaluated in terms of median survival time [9]. These findings were confirmed by Hou and coworkers who showed decreased miR-146a expression in 84% of cases and association of low miR-146a expression with reduced overall survival [39]. The link between miR-146a and gastric cancer is also related to the possible effects of this miRNA on the inflammatory response of Helicobacter pylori, a major human pathogenic bacterium in gastric mucosa. Particularly, Helicobacter pylori induces miR-146a expression through a NF-kB-dependent mechanism: in turn, miR-146a downregulates the expression of target genes, including IRAK1, TRAF6, IL-8 and, through this mechanism, modulates the inflammatory response [40,41].

miR-146a expression was studies in breast cancer. Constitutive NF-kB activity was frequently observed in breast cancer and was associated with aggressive breast cancer clinical behavior. Using the highly metastatic breast cancer cell line MDA-MB-231, exhibiting spontaneous NF-kB activation, Bhaumik et al showed that enforced miR-146a expression inhibited endogenous NFkB expression/activity and reduced the metastatic potential of these tumor cells [42]. A second study showed that the breast cancer metastasis suppressor 1 (BRMS1) could act by upmodulating miR-146a levels [7]. BRMS1 is a metastasis suppressor that affects multiple steps in the metastasis cascade of breast carcinoma, acting through different mechanisms which involve various genes whose function is related to the control of the metastatic potential. Thus, it was shown that BSMS1 markedly activates both miR-146a and miR-146b expression in breast carcinoma cells and, through this mechanism, inhibits EGFR expression, thus leading to a reduced metastatic potential of these cells [7].

Other studies suggest a possible role for miR-146a in prostate cancer. Lin et al performed a screening of miRs exhibiting a differential expression in androgen-independent as compared to androgen-dependent prostate cancer cell lines. Among the 8 miRNAs downregulated in androgen-resistant cell lines, compared to androgen-dependent cell lines, there were miR-146a and miR-146b [10]. In situ hybridization analysis of miR-146a expression in tissue prostate cancer sections showed that this miR is highly expressed in normal prostate tissue and significantly downmodulated in prostate cancer tissue [10]. Transfection of miR-146a in an androgen-independent prostate cancer cell line resulted in a marked reduction of cell proliferation, invasion and metastasis to bone marrow, an effect seemingly mediated by the targeting of ROCK1, one of the key kinases for the activation of hyaluronan [10].

Recent studies indicate that a deregulated miR-146a expression could contribute to glioma development through the targeting of NOTCH1, a cell signaling pathway playing a key role in the mechanism of neural stem cell development and maintenance in the adult life. Particularly, studies carried out in human glioblastomas have shown that miR-146a expression is upregulated in the tumor tissue, compared to the normal neural tissue [43]. In spite this enhanced expression in glioblastomas, experiments carried out in models of experimental gliomagenesis have shown that EGFR stimulation and PTEN inactivation induces miR-146a expression, that in turn acts as a mechanism limiting glioma development, through NOTCH1 targeting [11]. In particular, miR-146a plays an inhibitory role by restricting the formation of glioma stem-like cells and reducing the proliferation of glioma cells [11]. These observations suggest that miR-146a constitutes an endogenous feedback mechanism to counteract the oncogenic potential of dysregulated signaling pathways [11].

A G > C polymorphism in the miR-146a precursor sequence was shown to be positively associated with risk of developing glioma and with clinical prognosis in adult glioma patients [44].

Wang and coworkers have explored the pattern of miRNAs expression in cervical cancer, compared with normal cervical tissue. Several miRNAs appeared to be deregulated in their expression and, among them, miRNA-146a was found to be 10 fold upmodulated in cancer tissue compared to normal cervical tissue [45]. Surprisingly, cervical carcinoma cell lines were found to express miR-146a at very low levels and enforced expression of miR-146a in these cells caused a stimulatory effect on cell proliferation [45].

Recent studies on mouse miR-146a knockout models strongly supports a role for miR-146a as a tumor suppressor for myelo-lymphoid cells. In fact, the loss of miR-146a favors the development of myeloid and lymphoid neoplasia [22,23]. miR-146a KO mice developed at the age of 5-6 months a massive myeloproliferative disease. In fact, these mice showed a markedly enlarged spleen, massively infiltrated with myeloid blast precursors CD11b^+^GR1^+ ^cells [22]. Concomitantly, these mice displayed anemia, thrombocytopenia and lymphopenia, as well as extramedullary erythropoiesis [22]. A mild and variable expansion of CD11b^+ ^cells was observed also in the BM of miR-146a KO mice [22]. The myeloproliferation induced by miR-146a loss seems to be related to a deregulated activation of NF-kB [23] and the hyperexpression of M-CSFR in myeloid elements [22]. At later times in these KO animals often the myeloproliferation of spleen progresses up to the formation of splenic sarcomas and lymphomas of either B or T lineage are observed in older KO mice at a much higher frequency than in the normal WT animals [23].

A possible deregulation of miR-146 has been explored in various leukemic processes. Visone et al have explored the karyotype-specific miRNA signature in chronic lymphocytic leukemia (CLL) and found 9 miRNAs, including miR-146a and miR-146b, whose expression is deregulated in CLL compared to their normal counterpart [46]. The expression level of these miRs was correlated with gene expression data [46].

Several studies were devoted to the analysis of miR-146 expression in the various subtypes of acute myeloid leukemia (AML). Garzon et al performed a large screening on miRNA expression in 122 untreated AML samples, compared to normal CD34^+ ^cells [47]. miR-146a was among the miRs whose expression was most downmodulated, compared to normal CD34^+ ^cells [47].

Lutherborrow et al have compared the expression of some miRNAs, including miR-146a and miR-146b, in FAB M1 and FAB M5 AMLs [48]. In FAB M5 AMLs the expression of both miR-146a and miR-146b was lower than in FAB M1 AMLs; furthermore, miR-146a expression was significantly downmodulated during monocytic differentiation of AML cell lines induced by chemical inducers [48].

Spinello et al reported a detailed analysis of miR-146a expression in 38 untreated AML samples and observed heterogeneous levels for each FAB subtype, with the mean lowest levels observed in FAB M5 AML samples; furthermore, in many AML samples mR-146a levels were significantly lower than those observed in normal CD34^+ ^cells [49]. In parallel, it was investigated the level of one of the miR-146a targets, i.e., CXCR4: thus, CXCR4 protein levels were found to be inversely related to the levels of miR-146a, the highest CXCR4 levels being observed among FAB M5 AMLs [49]. Induction of differentiation of leukemic monocytic cell lines, such as U937, using a chemical inducer such as 1alpha25OH-VitD3, determines an upmodulation of miR-146a expression, accompanied by a concomitant reduction of CXCR4 protein levels. Importantly, enforced expression of miR-146a in leukemic cell lines determines a reduced cell proliferation and an increased sensitivity to the cytotoxic effects of anti-leukemic drugs [49].

Starczynowski et al have explored miR-146a expression in CD34^+ ^cells derived from 54 primary AML samples of patients with normal karyotype and observed reduced levels in leukemic samples compared to their normal counterpart [50]. Experiments carried out in leukemic cell lines, such as HL60, and showing an increased miR-146a expression following treatment with demethylating agents suggests that epigenetic mechanisms could be responsible for the reduced expression of miR-146a observed in these AML samples [50]. Enforced expression of miR-146a in these leukemic cell lines resulted in a clear inhibitory effect on cell proliferation [50].

Using the promyelocytic leukemia NB4 cell line, Zhang et al observed a downmodulation of miR-146a during ATRA (All Trans Retinoic Acid)-induced granulocytic differentiation, associated with upregulation of Smad4 protein levels [50]. According to these findings it was suggested that a role for the miR-146a/Smad4 regulatory pathway in retinoic acid-induced differentiation of APL cells [51].

In some myelodysplastic syndromes a genetic mechanism is directly responsible for the low expression of miR-146a. In fact, in the myelodysplastic 5q^- ^syndrome, the deletion of chromosome 5q correlates with the loss of two miRNAs, miR-145 and miR-146a [26]. Functional experiments have shown that the loss of these two miRs is responsible for main features of the 5q^- ^syndrome, such as neutropenia and thrombocytosis [26]. Other studies have confirmed decreased miR-146a levels in CD34^+ ^cells of the 5q^- ^myelodysplastic syndrome compared to the levels observed in normal CD34^+ ^cells [52]. However, surprisingly, normal miR-145 levels have been observed in CD34^+ ^5q^- ^MDS cells [52]. Particularly, although four miRNAs (miR-143, miR-145, miR-146a and miR-378) were mapped within the 5q deletion, only two of them, miR-146a and miR-378 showed reduced expression in 5q^- ^patients [52]. The non reduced miR-145 levels observed in CD34^+ ^cells could be explained by the recent finding that p53 transcriptionally induces miR-145 by interacting with a p53 response element present in the miR-145 promoter [53]. In fact, following the del (5q) one allele of miR-145 is lost, while the remaining allele could be transcriptionally hyperactive in consequence of p53-mediated stimulation.

An important issue in the analysis of the effects of miR-145 and miR-146 loss on the development of the myelodysplastic syndrome consisted in the study of irradiated mice transplanted with mouse stem/progenitor cells stably knocked down for miR-145 and miR-146 expression by retroviral-mediated overexpression of miR-145 and miR-146 target sequences (miR decoy). These mice initially developed a myelodysplastic syndrome, followed at distance of several months by the development of an acute myeloid leukemia [47].

However, the exact role and the contribution of miR-145 and miR-146 loss in 5q^- ^syndrome need to be carefully assessed in future studies [54].

MiR-146a expression was explored in chronic myeloid leukemia (CML) and, particularly, the changes in its expression occurring after treatment with imatinib. In untreated CML cells miR-146a levels were found to be significantly decreased compared to appropriate normal controls [55]. Interestingly, following treatment with imatinib miR-146a levels, as well as miR-150 levels, progressively increased up to restore normal levels of expression after 14 days of treatment with the tyrosine kinase inhibitor [55]. According to these findings it was suggested that the decreased miR-146a expression observed in untreated CML cells could contribute to mediate the BCR/ABL-induced NFkB constitutive activation; on the other hand, the increased miR-146a expression observed following imatinib treatment, could contribute to mediate the imatinib-induced NFkB inhibition [55].

A number of recent studies has shown a deregulation of miR-146a expression in some peculiar types of lymphomas. Among them particularly interesting are the observations made about miR-146a in NK/T cell lymphomas. The study carried out in this lymphoma has lead to the conclusion that miR-146a down-regulates NFkB activity via targeting TRAF6 and functions as a tumor suppressor having strong prognostic implications in NKT lymphomas [56]. This conclusion was based on the basic observation that NKT cell lymphomas with low miR-146a expression have a poor prognosis compared to those with high miR-146a expression [56]. Furthermore, in vitro miR-146a overexpression in NKT cell lines inhibited proliferation, induced apoptosis, and enhanced chemosensitivity [56].

MiR-146 expression is deregulated in virus-induced lymphoid neoplasias. Human T lymphotropic virus type I (HTLV1) is the etiologic agent of a severe and fatal lymphoproliferative disease, mainly affecting CD4^+ ^T lymphoid cells, adult T acute leukemia. Pichler et al have explored the miRNA profile of expression in HTLV1-transformed cells and observed that several miRs, including miR-21, miR-146a and miR-155 are hyper-expressed in HTLV1-transformed cells [57]. The mechanisms of miR-146a upregulation seem to be related to a stimulatory effect exerted by Tax, via NF-kB-mediated transactivation through binding to a NF-kB response element present in the miR-146a promoter [57]. In a second study Tomita and coworkers modulated miR-146a levels in HTLV1-transformed cells and observed that miR-146a inhibition in these cells by a specific antagomir elicited an inhibitory effect on cell growth, and opposite effect was induced by miR-146a-enforced expression [58].

The Epstein-Barr virus (EBV) infection is associated with the development of some lymphoid and epithelial cancers. EBV infection gives rise to an initial alteration of lymphocyte gene expression, which determines a stimulation of cellular proliferation and changes in the differentiation program, in consequence of the transition of the virus through consecutive latency transcription programs. The EBV-induced proliferation program involves pronounced changes at the level of various miRNAs, including miR-146a [59,60]. In this context, two initial studies have shown that the EBV-encoded latent membrane protein 1 induces the expression of miR-146a via NF-kB [59,60]. It was suggested that the induction of miR-146a may play a role in the induction or in the maintenance of EBV latency through the modulation of innate immune responses to the virus-infected host cells. A more recent study showed that de novo infection of primary human B lymphocytes with EBV induced a transient initial miR-146a downmodulation, followed by a pronounced upregulation more than 100 fold upon induction of the viral lytic cycle: this strong miR-146a induction seems to have inhibitory effects on the progression of the virus lytic cycle [60].

## Conclusion and future perspectives

The discovery of miRNAs as important regulators of gene expression shed a new light on our understanding of many biological processes. From the studies carried out during these last years it became evident miR-146a plays a key role as a modulator of the innate immune response. However, recent studies indicate also an important role of this miRNA in the control of normal hematopoiesis, particularly for that concerns the megakaryocytic and monocytic lineages. However, the mechanism, either direct or indirect through which miR-146a affects megakaryocytopoiesis and monocytopoiesis is still debated and additional studies are required to solve this issue.

On the other hand, growing evidence indicates a deregulation of miR-146a in many neoplastic conditions. This is the case of 5q^- ^syndrome associated to myelodysplasia, where a deficiency of miR-146a is observed. The deficiency of miR-146a, together with deficiency of miR-145, seems to be responsible for abnormalities of thrombopoiesis observed in this disease. Although the mechanism through which miR-146a induces a dysmegakaryopoiesis in 5q^- ^syndrome remains still unclear, knockout studies of the miR-146a locus indicate that reduced miR-146a expression affects hematopoiesis and contributes to features of myelodysplasia and leukemia. In line with these indications miR-146a expression is clearly downmodulated in many AMLs may contribute to the malign phenotype. Furthermore, miR-146a expression is deregulated in many solid tumors and there is consistent evidence that miR-146a may act as a tumor suppressor. However, the mechanism through which a miR-146a downmodulation may favor tumor development and/or maintenance is at the moment unclear, but it seems related to the capacity of this miRNA to target some mRNAs, such as TRAF6, IRAK1, CXCR4, EGFR. Future studies will unveil the molecular mechanisms through which miR-146a contributes to tumor development.

## Competing interests

The authors declare that they have no competing interests.

## Authors' contributions

CL and UT have equally contributed to the analysis of the literature data, to the redaction of the manuscript and its critical reviewing. All authors read and approved the final manuscript.
